# The influence of high myopia-related exotropia on surgical dose–response in unilateral recession and resection surgery: a retrospective study

**DOI:** 10.3389/fmed.2025.1588698

**Published:** 2025-06-30

**Authors:** Chao Li, ZhiYin Lou, Fan Yang

**Affiliations:** Joint Shantou International Eye Center of Shantou University and The Chinese University of Hong Kong, Shantou, China

**Keywords:** exotropia, high myopia, recession, resection, surgery

## Abstract

**Objective:**

To investigate the impact of high myopia on the surgical dose–response in unilateral recession and resection (R&R) surgery for exotropia.

**Methods:**

A retrospective analysis was conducted on 101 Chinese patients who underwent unilateral R&R surgery for concomitant exotropia between June 2021 and June 2024. Patients were divided into two groups based on axial length: those with high myopia (*n* = 38) and those without high myopia (*n* = 63). Surgical doses for lateral rectus (LR) recession and medial rectus (MR) resection were compared between the groups. Preoperative and postoperative deviations were measured, and statistical analyses were performed to assess the relationship between surgical dose, axial length (AL), and other clinical variables.

**Results:**

Age, duration of exotropia, and AL differed significantly between the groups, but other baseline characteristics were comparable. The LR recession dose did not differ significantly between the high myopia group (6.40 ± 1.11 mm) and the non-high myopia group (6.74 ± 1.00 mm, *p* > 0.05). However, the MR resection dose was significantly lower in the high myopia group (5.77 ± 1.02 mm) compared to the non-high myopia group (6.32 ± 0.92 mm, *p* < 0.05). The MR resection and LR recession dose was positively correlated with the horizontal deviation (*p* < 0.001), but showed no significant correlation with AL (*p* > 0.05).

**Conclusion:**

Exotropia with high myopia had a greater surgical dose response than those without high myopia. No significant difference was found in LR recession dose, but the MR resection dose was significantly lower in the high myopia group.

## Introduction

Exotropia, a form of strabismus characterized by outward deviation of the eye, is a condition involving abnormal ocular alignment (1). Despite the higher prevalence of esotropia in Western populations, epidemiological studies have demonstrated that exotropia is more frequently observed than esotropia in Asian populations (2). Exotropia not only impacts patients’ binocular vision but also impairs their ability to perform daily activities and has adverse effects on their overall health and quality of life (3). Exotropia frequently co-occurs with myopia. Studies have reported that the prevalence of myopia in Australian children aged 12 years with exotropia is as high as 57.7%, significantly higher than the 12.3% observed in children without exotropia (4). Additionally, the Multiethnic Pediatric Eye Disease Study (MEPEDS) in Southern California and the Baltimore Pediatric Eye Disease Study (BPEDS) in Maryland, which examined children aged 6 to 72 months, found a higher prevalence of myopia in children with exotropia (12.7%) compared to those without exotropia (4.6%) (5). Related studies (6) have also found that myopia significantly increases the risk of exotropia. These findings highlight the association between exotropia and myopia. Over the past few decades, the incidence of myopia has been increasing, with a notable rise in the prevalence of high myopia, particularly in East Asian populations (7).

The treatment of exotropia primarily includes both surgical and non-surgical methods, with surgery being a common intervention. Surgical approaches typically involve bilateral lateral rectus (LR) muscle recessions or unilateral LR muscle recession combined with medial rectus (MR) muscle strengthening. Some surgeons prefer bilateral surgery when the distance deviation exceeds the near deviation, whereas unilateral surgery is favored when the near deviation is greater than the distance deviation. These decisions are often guided by the specific characteristics and severity of the patient’s exotropia (8). Relevant studies have suggested that the success rate of unilateral recession and resection (R&R) surgery is significantly higher compared to bilateral lateral rectus recession (BLR) surgery. Patients who undergo BLR surgery are more likely to experience relapse. However, no significant difference was observed in the overcorrection rate between BLR and R&R procedures (9). These findings indicate that unilateral R&R surgery may be a more effective and stable option for the treatment of exotropia.

The study has reported that the axial length (AL) is not related to the dose of exotropia surgery (10). However, Beisse et al. (11) observed a negative correlation between AL and the surgical dose response. The current research findings in this area remain contradictory. In clinical observations, we noted that patients with high myopia (HM) appear to require less surgical intervention in concomitant exotropia surgery. This observation prompted the hypothesis that high myopia may influence the amount of surgical dose needed, potentially due to anatomical changes in the eyeball associated with high myopia.

Accurate prediction of the surgical dose response is essential for determining the optimal surgical dose in strabismus surgery to achieve satisfactory outcomes. Therefore, in this study, we aimed to compare the differences in the surgical dose of R&R surgery among various exotropia groups, providing a dose–response reference for patients with different AL variations.

## Methods

This retrospective study was approved by the Review Board of the Joint Shantou International Eye Center of Shantou University and The Chinese University of Hong Kong, and adhered to the principles of the Declaration of Helsinki.

We reviewed the medical records of patients who underwent unilateral R&R surgery for concomitant exotropia at the JSIEC between June 2021 and June 2024. Patients were included if they achieved successful motor alignment, defined as a deviation of no more than 10 prism diopters in primary gaze at both 33 cm and 6 meters.

Inclusion criteria: patients diagnosed with exotropia (include constant exotropia and intermittent exotropia) who exhibited no motility restrictions and whose deviation in gaze direction was essentially constant. For patients in the high myopia group with concomitant exotropia, the additional criterion was an AL ≥ 26 mm (12). Exclusion criteria: patients with paralytic or restrictive strabismus; a history of previous horizontal strabismus surgery or botulinum toxin injections; concomitant vertical repositioning of the horizontal rectus muscle insertions or surgical interventions on the vertical rectus muscles were performed to address vertical deviations. Head trauma; and any other ophthalmologic or systemic neurological conditions that could affect eye movement. We included patients who underwent simultaneous inferior oblique muscle weakening procedures for treating inferior oblique overaction, as prior studies have shown that this intervention does not significantly impact horizontal alignment in the primary position (13, 14).

All patients underwent comprehensive ophthalmic examinations, which included measurement of best-corrected visual acuity using a standard logarithmic visual acuity chart, the Titmus stereoacuity test, the Worth 4-dot test for fusion assessment, and a complete slit-lamp examination with fundus evaluation. AL was measured using the OA 2000 in all patients. Binocular alignment was assessed using the alternate prism cover test at both near (33 cm) and far (6 m) distances with full refractive correction. Neuroimaging was performed in cases where neurological abnormalities were present to rule out intracranial pathology.

The preoperative horizontal deviation was re-measured on the day before surgery, and all procedures were performed by the same ophthalmologist. Based on our clinical experience, postoperative overcorrection is common in patients with concomitant exotropia and high myopia. According to the surgeon’s surgical table, we reduced the dose of unilateral RR surgery for patients with concomitant exotropia and high myopia, while using the conventional dose (Table 1) for patients without concomitant exotropia. All surgical procedures were performed under general anesthesia, regardless of the patient’s age. No adjustable sutures were used in any of the patients.

**Table 1:** Conventional surgical dose.

Deviation (PD)	LR recession (mm)	MR resection (mm)
20	4	4
25	5	4
30	6	4.5
40	7	5.5
50	7.5	6
60	8	6.5
70	8	7.5
80	8	8

LR, Lateral Rectus; MR, Medial Rectus; Deviation, calculated as the average of distant and near horizontal angles. The surgical dose for LR recession will refer to the average horizontal deviation and the distant horizontal deviation; the surgical dose for MR resection will refer to the average horizontal deviation and the near horizontal deviation.

Refractive errors were converted to spherical equivalent for all patients and calculated as the algebraic sum of the diopters of the spherical component and half the cylindrical component.

### Statistical analysis

All data analyses were performed using R software (version 4.3.3, R Foundation for Statistical Computing, Vienna, Austria). To evaluate baseline differences between the two groups, an independent t-test was employed for normally distributed continuous variables, while the Kruskal-Wallis H test was utilized for non-normally distributed continuous variables. Categorical variables were compared using the Chi-Square test. For the primary outcome, a general linear model was applied to assess differences in the amount of MR resection and LR recession surgery between the groups. In unadjusted models, the categorical predictor was group. Adjusted models included other clinical variables as covariates to control for baseline differences, such as age, sex, duration of strabismus, axial length, and the presence of amblyopia and anisometropia. Statistical significance was defined as a two-sided *p* value of less than 0.05.

## Result

This study included 38 exotropia patients with high myopia and 63 exotropia patients without high myopia, all of whom were of Chinese ethnicity and underwent unilateral R&R performed by the same surgeon. Baseline demographic and clinical characteristics, including gender (exotropia without HM:33/30, exotropia with HM:22/16), anisometropia (exotropia without HM:49/14, exotropia with HM:25/13), amblyopia (exotropia without HM:62/1, exotropia with HM:35/3), operated eye (exotropia without HM:25/38, exotropia with HM:19/19), and IO weakening (exotropia without HM:56/7, exotropia with HM:30/8), were comparable between the two groups (all *p* > 0.05). However, significant differences were observed in age (exotropia without HM:11.32 ± 4.52, exotropia with HM:15.05 ± 5.19), duration of exotropia (exotropia without HM:3.35 ± 2.73, exotropia with HM:5.58 ± 4.77), and AL (exotropia without HM:23.79 ± 0.92, exotropia with HM:27.00 ± 1.06) (*p* < 0.05). Preoperative deviation angles, measured at near (exotropia without HM:58.43 ± 16.38, exotropia with HM:63.32 ± 18.52), distance (exotropia without HM:39.52 ± 11.73, EXO with HM:39.08 ± 12.51), and the average (exotropia without HM:48.98 ± 12.70, exotropia with HM:51.20 ± 14.26) of near and distance, showed no statistically significant differences between the two groups (all *p* > 0.05) (Table 2).

**Table 2:** Base characteristics of participants.

Characteristic	EXO without HM (*N* = 63)	EXO with HM (*N* = 38)	*p*
Age (years)			<0.001^a^
Mean ± SD	11.32 ± 4.52	15.05 ± 5.19	
Range	[5 to 34]	[7 to 26]	
Gender			0.590^b^
Male	33	22	
Female	30	16	
Duration (years)			
Mean ± SD	3.35 ± 2.73	5.58 ± 4.77	0.014^a^
Range	[1 to 12]	[1 to 19]	
Anisometropia			0.187^b^
Without	49	25	
With	14	13	
Amblyopia			0.115^b^
Without	62	35	
With	1	3	
Operated eye			0.311^b^
Right	25	19	
Left	38	19	
IO weakening			0.173^b^
Without	56	30	
With	7	8	
AL (mm)	23.79 ± 0.92	27.00 ± 1.06	<0.001^c^
Range	[21.92 to 25.98]	[26.02 to 31.15]	
Horizontal angle in primary position (PD)			
Distant	39.52 ± 11.73	39.08 ± 12.51	0.791^a^
Range	[15 to 60]	[20 to 60]	
Near	58.43 ± 16.38	63.32 ± 18.52	0.164^a^
Range	[25 to 85]	[30 to 86]	
Average of distant and near	48.98 ± 12.70	51.20 ± 14.26	0.477^a^
Range	[20 to 72.50]	[27.50 to 73]	

EXO, Exotropia; HM, High Myopia; PD, Prism Diopters.

a Kruskal–Wallis H test.

b Chi-Square test.

c Independent *t* test.

**Figure 1:**
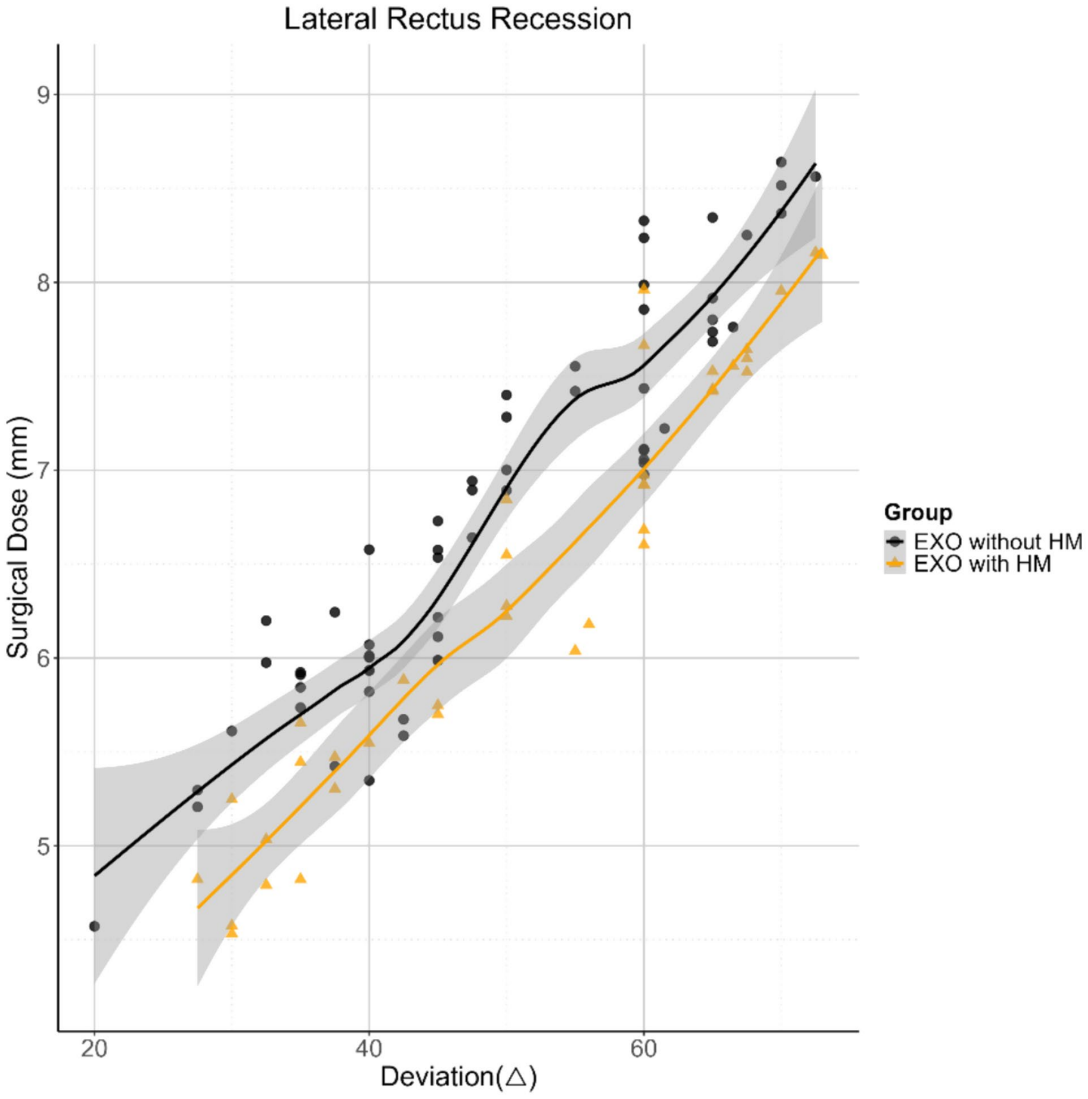
Scatterplots with smooth fit lines depicting the relationship between horizontal deviation and the surgical dose of lateral rectus recession in both groups. EXO, exotropia; HM, high myopia.

In all three models, LR recession dose did not differ significantly between the exotropia with HM and exotropia without HM groups. The LR recession dose demonstrated a significant positive correlation with the horizontal deviation angle (average of distant and near measurements; *p* < 0.001). However, no significant correlations were observed between the LR recession dose and other clinical variables, including age, gender, duration of exotropia, anisometropia, amblyopia, operated eye, IO weakening, or AL (all *p* > 0.05). To account for potential baseline imbalances between groups, adjustment models were constructed, incorporating age, gender, duration, anisometropia, amblyopia, operated eye, IO weakening, AL, and horizontal deviation. Figure 1 illustrates the relationship between the LR recession dose and horizontal deviation after model adjustment (Table 3).

**Table 3:** General linear model.

Surgery	Model 1		Model 2		Model 3	
	Mean difference	*p*	Mean difference	*p*	Mean difference	*p*
LR recession (mm)						
EXO without HM	6.73 ± 0.95	0.163	6.73 ± 0.17	0.052	6.74 ± 1.00	0.295
EXO with HM	6.43 ± 1.16		6.43 ± 0.19		6.40 ± 1.11	
MR resection (mm)						
EXO without HM	6.29 ± 0.86	0.017	6.29 ± 0.22	0.004	6.32 ± 0.92	<0.001
EXO with HM	5.80 ± 1.17		5.81 ± 0.22		5.77 ± 1.02	

Model 1: Unadjusted model; Model 2: Adjusted for age, gender; Model 3: Adjusted for age, gender, duration, anisometropia, amblyopia, operated eye, IO weakening, AL and horizontal deviation (average of distant and near).

**Figure 2:**
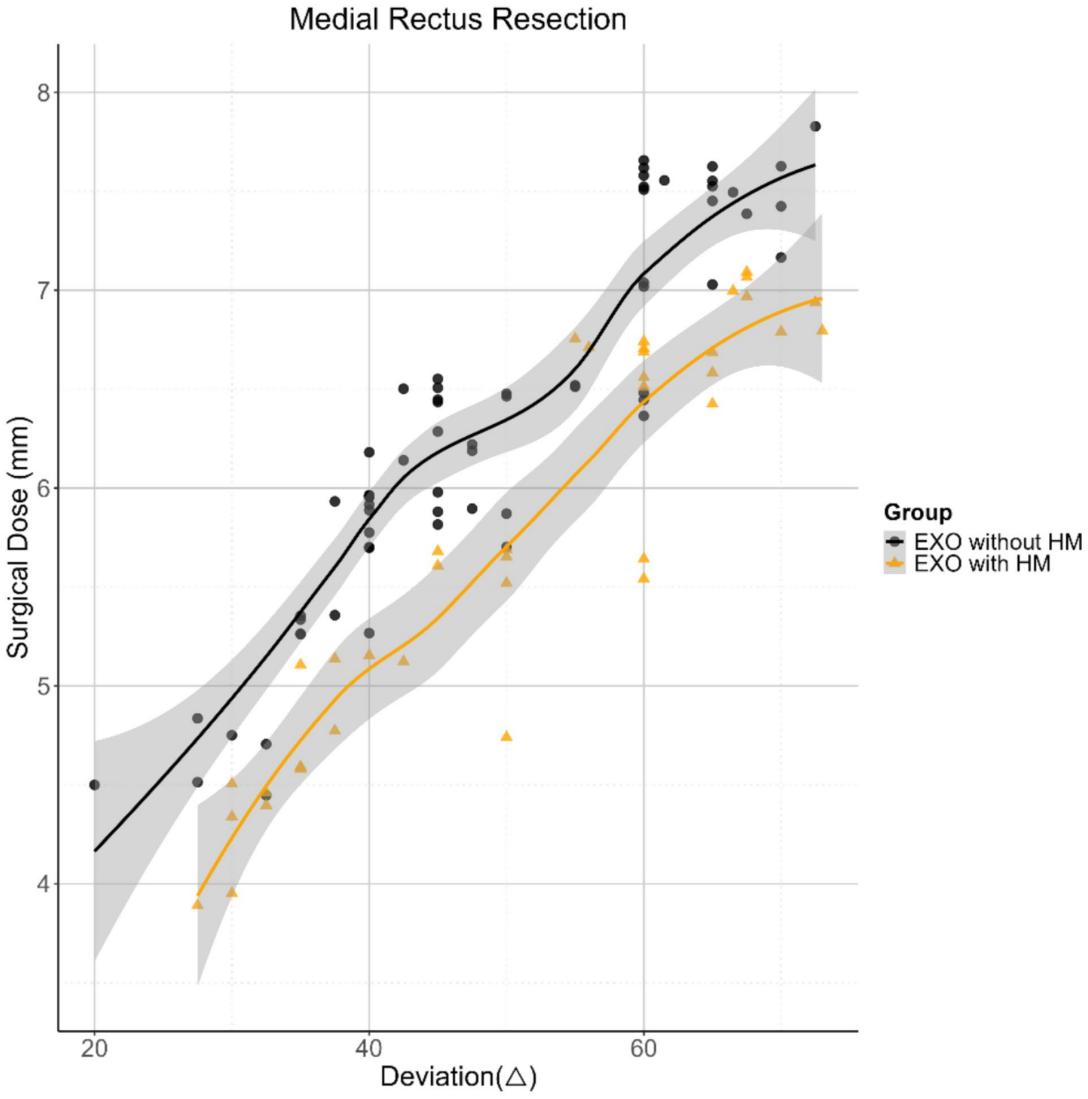
Scatterplots with smooth fit lines depicting the relationship between horizontal deviation and the surgical dose of medial rectus resection in both groups. EXO, exotropia; HM, high myopia.

In contrast, the MR resection dose differed significantly between the exotropia with HM and exotropia without HM groups across all three models (*p* < 0.05). Specifically, in Model 3, the MR resection dose was significantly lower in the exotropia with HM group compared to the exotropia without HM group. The MR resection dose was positively correlated with the horizontal deviation angle (average of distant and near measurements; *p* < 0.001). No significant correlations were found between the MR resection dose and gender, duration of exotropia, amblyopia, operated eye, IO weakening, or AL (all *p* > 0.05). However, a negative correlation was observed between the MR resection dose and anisometropia (*p* = 0.018). Adjustment models were similarly applied to control for baseline imbalances, incorporating the same clinical variables. Figure 2 depicts the relationship between the MR resection dose and horizontal deviation after model adjustment (Table 3).

## Discussion

This retrospective study analyzed disparities in surgical dosage between patients with and without high myopia following successful unilateral R&R surgery for exotropia. By evaluating dose–response relationships and quantifying the magnitude of deviation in MR resection and LR recession, we proposed a calculation method to tailor surgical dosing according to individual patient characteristics.

Postoperative deviation angles were assessed at the first postoperative month to evaluate the efficacy of unilateral R&R surgery. Early postoperative measurements (within 1 week) may be confounded by perioperative factors such as patient discomfort, incomplete tissue healing, or transient convergence dysfunction, potentially leading to unreliable results (15). Furthermore, the gradual onset of exodeviation—a recognized phenomenon following exotropia surgery—necessitates a balanced timeframe for assessment (16). The one-month postoperative interval minimizes the influence of acute postoperative variables while mitigating the progressive effects of exodeviation, thereby providing a more accurate and clinically relevant reflection of the unilateral R&R procedure’s surgical outcome (17).

Investigations into the correlation between age at exotropia surgery and surgical response have yielded inconsistent findings, with some results appearing to be contradictory. Related research (18) has demonstrated a significant negative correlation between the surgical response to exotropia and the age at which surgery is performed (*p* < 0.01). In patients older than 12 years, increasing the surgical dose resulted in a significant improvement in the success rate compared with the group that did not have an increase in surgical dose (80% vs. 41%). However, other studies (19, 20) have proposed that there is no significant association between surgical age and surgical response. A retrospective study (17) involving 295 Chinese patients with exotropia demonstrated no significant impact of age at surgery on the surgical dose–response relationship within the pediatric cohort. Conversely, in the adult group, a downward trend in the surgical dose–response was observed among patients aged over 35 years. It is noteworthy that our study found no correlation between age at surgery and surgical response, and all subjects included in our study were under the age of 35 years.

However, the results of this study showed that patients with exotropia and high myopia (AL ≥ 26 mm) had a lower exotropia surgical dose than patients with exotropia and non-high myopia. In addition, this study also found that there was no linear correlation between the dose of exotropia surgery and the AL, which was similar to the conclusions of the current studies (10, 21).

**Figure 3:**
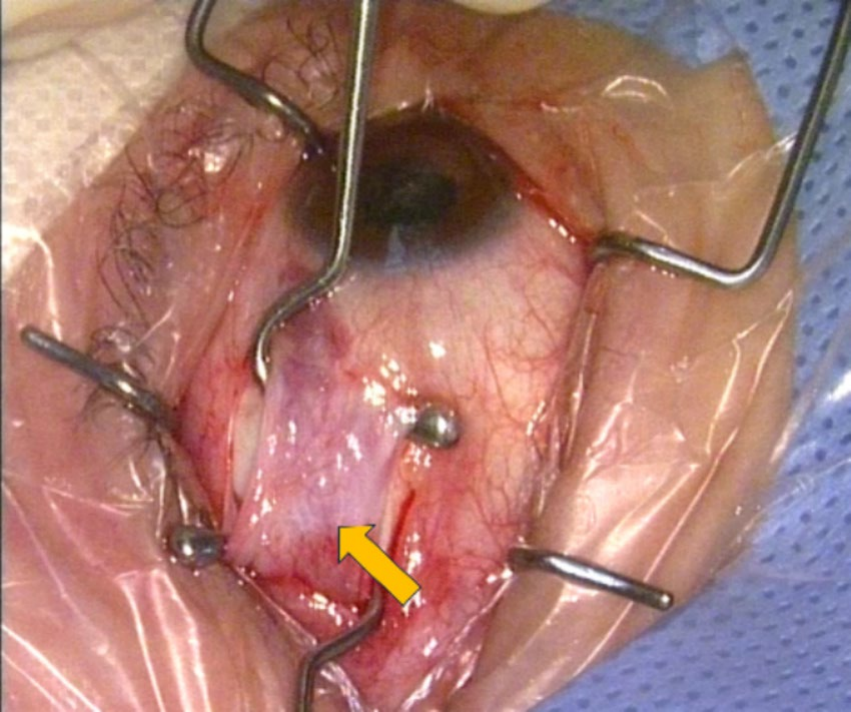
The yellow markings denote the presence of fibrosis in the medial rectus muscle.

This phenomenon suggests that the low surgical dose in patients with exotropia and high myopia may be affected by many factors. A hypothesis (17) has been proposed that in eyes with long AL, the resection of extraocular muscles by MR resection may exceed the limit of linear proportional response, resulting in an increased response. This situation is analogous to a force applied to a spring that exceeds the elastic reaction region and no longer follows Hooke’s law. During the operation of this study, fibrosis of the medial rectus muscle was also found in some patients with exotropia and high myopia (Figure 3), which may be due to changes in the elasticity of the MR, resulting in a more significant response to the surgical dose.

In addition, relevant study (22) has found that the overall median thickness of the MR in myopic patients is significantly smaller than that in emmetropic patients, and the parameters of the MR (thinner and smaller) are significantly correlated with myopia. However, this trend has not been observed in the LR. In this study, there was no statistically significant difference in the surgical dose of LR recession between the exotropia with high myopia group and the exotropia without high myopia group, while the surgical dose of MR resection was less in the exotropia with high myopia group than in the exotropia without high myopia group.

This study has several limitations that should be acknowledged. First, it was a single-center, single-race, retrospective study with a relatively small sample size. Future studies should involve larger cohorts, multiple medical centers, and a diverse range of ethnic groups to confirm and refine the current findings. Second, postoperative strabismus deviation was measured 1 month after surgery. This time frame may not fully capture the long-term outcomes of the surgical intervention. Third, we defined high myopia based on AL rather than refractive error. AL was chosen as the criterion because it is a more stable parameter, less susceptible to accommodative influences, and more accurately reflects the mechanical and biological characteristics of the eye. Fourth, the mechanisms underlying the greater surgical dose response in patients with high myopia were not investigated in this study. Further research is planned to explore these mechanisms. Fifth, patients undergoing large-angle bilateral exotropia correction surgery were not included in this study. These cases will be examined in future studies.

The surgical dose response in patients with exotropia and high myopia was found to be greater than that in patients with exotropia without high myopia. Specifically, there was no significant difference in the surgical dose of LR recession between the exotropia with high myopia group and the exotropia without high myopia group. However, the surgical dose of MR resection was significantly lower in the exotropia with high myopia group compared to the exotropia without high myopia group.

## Data Availability

The original contributions presented in the study are included in the article/supplementary material, further inquiries can be directed to the corresponding author.
